# Modern Flow Analysis

**DOI:** 10.3390/molecules25122897

**Published:** 2020-06-24

**Authors:** Paweł Kościelniak

**Affiliations:** Faculty of Chemistry, Jagiellonian University, Gronostajowa 2, 30-387 Krakow, Poland; pawel.koscielniak@uj.edu.pl; Tel.: +48-1268-62411

**Keywords:** flow analysis, flow chemistry

## Abstract

A brief overview of articles published in this Special Issue of Molecules titled “Modern Flow Analysis” is provided. In addition to cross-sectional and methodological works, there are some reports on new technical and instrumental achievements. It has been shown that all these papers create a good picture of contemporary flow analysis, revealing the most current trends and problems in this branch of flow chemistry.

The idea to process an analytical sample through its flow to a measuring apparatus was one of the milestones in the development of the chemical analysis. The creator of this idea is considered Leonard T. Skeggs, Jr., who was the first to construct an analytical flow system and in 1957 presented its application in clinical analysis [1]. The motive for his action, as he wrote himself, was quite simple: “The staffs of laboratories of clinical chemistry are confronted with an ever-increasing number and variety of determinations”, and it is also current, maybe even more so nowadays. Therefore, it is no wonder then that flow analysis has grown over the years and has found more and more followers.

Since "Skeggs times", many different flow techniques, systems and modules have been proposed, providing the possibility of analyzing many samples in a short time with increased safety of analytical work and with results of very good sensitivity and precision. These fully mechanized, automated and often miniaturized systems are increasingly using new methodologies and materials to improve the quality and speed of sample processing. The flow mode has been shown to favor various ways of manipulating sample and reagents, facilitating analytical operations such as calibration, titration or multi-component analysis. It also turned out that the hydrodynamic conditions of the flowing solutions create new possibilities for conducting and controlling chemical reactions. 

The latter feature of flow systems has been noticed and exploited in other chemical fields, particularly in organic chemistry. Constructed slightly later than analytical systems, the first flow systems dedicated to the synthesis of organic compounds proved to be so useful and effective that, over the years, flow synthesis has acquired the established general name "flow chemistry", bypassing flow analysis. This situation makes it difficult to objectively assess the current state of flow analysis and its proper role and place in the development of chemistry as applied science.

Unfortunately, the aforementioned problem usually escapes the attention of flow analysts. More important and valuable is the review article written by Trojanowicz entitled “Flow Chemistry in Contemporary Chemical Sciences: A Real Variety of Its Applications” [2]. In this article, the author presents the in-depth comparative characteristics of flow analysis and flow synthesis, paying special attention to the chronology of inventions of physico-chemical operations and appropriate instrumental devices, which are widely employed in both areas of flow chemistry. It reveals many of their mutual, sometimes surprising similarities in terms of both the construction and operation of flow systems and their stages of development, primarily in the spheres of mechanization, automation and miniaturization. He proves his thesis with many literature examples (357 references!), including selected items from recent years, giving an excellent picture of contemporary, modern flow analysis and flow synthesis. He finishes his work with a very important statement: “...it seems to be fully justified to use the term flow chemistry to represent all other chemical processes carried out under flow conditions of the reacting mixture, a sample to be analyzed, and other media chemically transformed under flow conditions”. In my opinion, the article should be a must-read for analysts interested in flow chemistry, and above all for those who are starting their scientific and didactic work in this field.

A very good example of how to take advantage and practice the common features of flow chemistry is presented in [3]. The flow-based system working on the principle of segmented injection analysis (SIA), very popular and currently widely used in flow analysis, was used for synthesis of uniform micron-size CS-Cu(II) catalyst particles. It was also exploited to fast and effective monitoring of the catalytic activity of the synthesized particles for the reduction of p-nitrophenol with excess borohydride. The flow-based method provided advantages over the manual method in terms of throughput for preparation of the particles (100 drops min^−1^), size distribution of the particles (150–210 µm) and their uniformity.

Another example justifying the concept of flow chemistry are the flow-based studies of bioaccessibility of bioactive compounds from food. The bioaccessibility test provides valuable information to choose the right dose and source of food matrices and thus to ensure the nutritional efficiency of food products. A valuable advantage of flow systems is the ability to quickly and precisely test the rate of absorption of compounds. Such a system was used for the implementation of dynamic extractions, aiming at the evaluation of bioaccessible zinc and the characterization of leaching kinetics in dry dog food samples [4]. This dynamic procedure was proved to be more flexible than the static traditional batch methods, allowing, in addition, the natural non-equilibrium processes to be much better imitated.

Dynamic flow procedures are also the main subject of the review paper written by Qiao [5]. In this case, the author focused on the implementation of various flow techniques for the determination of radionuclides. Typical analytical challenges involved in this area include, for instance, very low radioactivity of a sample, matrix effects and need to separate exactly the radioisotopes from the sample matrix. Based on the literature study author proved that the flow analysis meets all these requirements. He showed that the versatile flow approaches can be utilized in different steps for radiochemical analysis, including sample pretreatment, chemical separation and purification, as well as source preparation and detection. The flow mode makes the analysis fast, low laborious and-what is perhaps most important in this case-safe for operators because of less exposure to radioactivity. In conclusion, the author stated that “...continuous development of more advanced flow approaches is necessary to cope with the growing demands for radiochemical analysis in different fields...”.

As if in response to this expectation, in [6], a commercially available fully automated flow-based device (SeaFAST) is presented that is dedicated to the determination of ^90^Sr at trace levels in nuclear spent fuel leachate samples. Isotope ^90^Sr is a fast released and hard to measure fission product and is of great interest due to its toxicity and high energy emission. The system, composed of an autosampler, series of syringes and a valve module, is coupled to ICP-MS combines the use of a Sr-specific resin and the reaction with oxygen as reaction gas in a dynamic reaction cell (DRC). As experimentally proved, strontium was possible to be determined in a single operational sequence of separation, pre-concentration and elution avoiding sophisticated and time-consuming procedures. In addition, the developed method was revealed to be safe, rapid, selective, and sensitive, showing a good agreement in terms of measurement uncertainties when compared with the classical radiochemical method. 

Syringe-based flow systems are one of the examples of very useful and efficient flow devices that provides the possibility to establish different sample pathways and to assure a very stable and reproducible flow rate. Multisyringe Flow Injection Analysis (MSFIA) has been introduced in 1999 [7] and was intensively developed in the following years. In 2012 it was shown that the syringe can be used not only as a kind of pump, but also as a chamber in which the sample is fully processed before measurements [8]. From now on, this technique, called Lab-in-Syringe (LIS), is gaining more and more interest, constantly undergoing new modifications, modes of operation and technical improvements.

In this context, a very needed and helpful article is work [9], aimed to bring the LIS technique closer to newcomers and users of other flow techniques. The article reviews the different options for instrumental configurations and operations possible to be performed using LIS, including syringe orientation, in-syringe stirring modes, in-syringe detection, additional inlets, and addable features. Great attention is paid to LIS applications in the field of automation of the sample pretreatment procedures, especially of extraction processes in the liquid phase. The possible contributions of 3D printing techniques to LIS are also mentioned. In addition to the unmistakable advantages of this technique, some of its limitations are discussed that arise mainly from the large dead volume of a syringe. The article as a whole is not only a very good guide to LIS, but also gives a more general picture of new conceptual, technical and instrumental tools contributing to the development of flow analysis.

A common feature of different flow techniques developed in the last years is the minimization of sample volume, reagent consumption and waste production. These features are consistent with the requirements of the recently fashionable and very needed policy named “green analytical chemistry” (GAC). Its purpose is obviously to reduce the risk from analytical laboratories to the environment and human health. Flow analysis naturally favors these aspirations, although different efforts are still underway to improve flow techniques in this direction.

An example of a GAC-oriented flow technique is the Sequential Injection Analysis (SIA) [10]. It consists of gradually introducing small segments of the sample and reagents into a separate conduit, mixing them and delivering to the detector after bringing the reaction to a certain stage. Such a technique, named zone fluidics by authors, was used for the spectrofluorimetric determination of histidine in the urine samples [11]. Before reaching the detector, the reacting sample was stopped for a time selected as a compromise between sensitivity and sampling throughput. The method allowed histidine to be determined directly with minimum sample preparation and with very good precision and accuracy. The method ensured also minimal consumption of reagents and generation of waste compared to continuous flow techniques.

The “green” idea can be also implemented by completely processing a sample with a very small volume using the selection valve (Lab-on-Valve), which is an integral part of the SIA system. Such a technique (µSIA-LOV) was applied to the determination of cholesterol in serum samples [12], which is a widely relevant in clinical diagnosis, since higher values of cholesterol in human blood are an important risk factor for cardiovascular problems. The analytical method was based on the implementation of enzymatic reactions performed in the µSIA-LOV system by cholesterol esterase, cholesterol oxidase and peroxidase. The results obtained were shown to be reliable and accurate, confirming the usefulness of this methodology for the routine determinations of cholesterol and for other clinical determinations. The automation and the miniaturization of the analytical procedure leads to the reproducibility improvement and the reduction of reagents. As the authors emphasize, the revealed advantages are relevant when the methodology developed is compared with other automatic methodologies used in the flow analysis.

The modification of the segmented injection analysis (SIA) towards GAC may also consist of merging the sample and reagents in the form of liquid segments limited by air segments of very small volume (on the order of several or several dozen microliters}. After some time, a single segment of reaction products is formed that is of limited dispersion and undiluted by the carrier stream. An example of the successful application of this mono-segmented technique is the simultaneous determination of albumin, glucose, and creatinine (the key biomarkers for diabetes mellitus) in the urine samples [13]. Due to the flow methodology, the fast reaction (for albumin) and slow reactions (for glucose and creatinine) were appropriately synchronized. From the analytical reliability point of view, what was important was the possibility of calibrating carefully the determinations by both external and standard addition methods without the change of the system configuration.

In work [14], the mono-segments were created for the titration purpose with use of three syringe pumps equipped with nine-position selection valves. During the titration process, two syringe pumps dispensed well-known decreasing and increasing volumes of the sample and titrant, respectively, which were then introduced simultaneously into the system, joined at the confluence point, and merged in the mixing coil to complete the reaction. Before and after introducing the defined volumes of sample and titrant, a segment of air was inserted using the third pump to form a monosegment. This simple and fully automated procedure, imitating traditional titration way, was used for the determination of iron(III) in the presence of iron(II), allowing one analysis to be performed for 6 min with very good precision and accuracy, and consuming as little as 2.4 mL of both sample and titrant solution.

Yet another approach to GAC requirements is to develop methods of sample processing that do not require a large number or amount of reagents. One of the ways used very often for the sample preparation is the sample preconcentration coupled with the analyte isolation from the sample matrix. It has been shown that a sample can be effectively preconcentrated in the mechanized sequential injection system on the basis of the membraneless evaporation [15]. The main element of the system was the preconcentration module working under high temperature and diminished pressure. Using different evaporation conditions, various values of the signal enhancement factor (from several to 20) could be achieved. The applicability of the method was positively verified on the example of the determination of Zn in certified reference materials of drinking water and wastewater using the capillary electrophoresis method. 

The purpose of sample preconcentration is to enhancement the analytical signal, which consequently gives the opportunity to determine the analyte with increased precision and diminished limit of quantification. The signal enhancement can be performed by various ways. A very interesting way has been displayed with the use of the simple flow manifold equipped with multicommutated solenoid valves and a spectrofluorimetric detector [16]. The analyte was online irradiated with UV light to produce a highly fluorescent photoproduct that was then retained on a solid support placed in the detector flow cell. By doing so, the pre-concentration effect of the photoproduct was achieved in the detection area, increasing the sensitivity. The method was demonstrated on the example of the determination of insecticide thiacloprid, one of the main neonicotinoids, in lettuce samples. The analytical results were characterized with very good precision and accuracy, and a low detection limit of 0.24 mg kg^−1^.

One more modification of the flow manifold that aimed to improve the detection capability is shown in [17]. This work presents a dual detector that consisted of a paired emitter–detector diode (PEDD) and a capacitively coupled contactless conductivity detector (C4D) for flow-based photometric and conductivity measurements, respectively. They have been incorporated in a single flow cell of an original design. In different flow configurations, the system was able to be adapted to either sequential or simultaneous determination of two analytes in a sample. In particular, the urine samples were analyzed in regard to the conductivity and creatinine concentration for monitoring the health problems in the human body.

It is seen that the articles included in this Special Issue of Molecules titled "Modern Flow Analysis" very well reflect the current state of flow analysis, which to a large extent is also a picture of modern flow chemistry. At the same time, these works give a picture of extraordinary ingenuity and creativity in solving problems and creating new, more and more perfect analytical approaches. Once again, it turns out that flow analysis is the area of analytical chemistry, in which imagination and scientific courage play a great role. One should hope that this is a guarantee of its further development in the future. 

## References

[1] Skeggs L.T. (1957). An automatic method for colorimetric analysis. Am. J. Clin. Path..

[2] Trojanowicz M. (2020). Flow chemistry in contemporary chemical sciences: A real variety of its applications. Molecules.

[3] Intanin A., Inpota P., Chutimasakul T., Tantirungrotechai J., Wilairat P., Chantiwas R. (2020). Development of a simple reversible-flow method for preparation of micron-size chitosan-Cu(II) catalyst particles and their testing of activity. Molecules.

[4] Gregório B.J.R., Pereira A.M., Fernandes S.R., Matos E., Castanheira F., Almeida A.A., Fonseca A.J.M., Cabrita A.R.J., Segundo M.A. (2020). Flow-based dynamic approach to assess bioaccessible zinc in dry dog food samples. Molecules.

[5] Qiao J. (2020). Dynamic flow approaches for automated radiochemical analysis in environmental, nuclear and medical applications. Molecules.

[6] Vilas V.V., Millet S., Sandow M., Pérez L.I., Serrano-Purroy D., Van Winckel S., Aldave de las Heras L. (2020). An automated SeaFAST ICP-DRC-MS method for the determination of ^90^Sr in spent nuclear fuel leachates. Molecules.

[7] Cerdà V., Estela J.M., Forteza R., Cladera A., Becerra E., Altimira P., Sitjar P. (1999). Flow techniques in water analysis. Talanta.

[8] Maya F., Estela J.M., Cerdà V. (2012). Completely automated in-syringe dispersive liquid-liquid microextraction using solvents lighter than water. Anal. Bioanal. Chem..

[9] Horstkotte B., Solich P. (2020). The automation technique Lab-in-Syringe: A practical guide. Molecules.

[10] Růžička J., Marshall G. (1990). Sequential injection: A new concept for chemical sensors, process analysis and laboratory assays. Anal. Chim. Acta.

[11] Alevridis A., Tsiasioti A., Zacharis C.K., Tzanavaras P.D. (2020). Fluorimetric method for the determination of histidine in random human urine based on zone fluidics. Molecules.

[12] Barbosa J.S., Passos M.L.C., Korn M.A., Saraiva M. (2020). Enzymatic reactions in a Lab-on-Valve system: Cholesterol evaluations. Molecules.

[13] Kiwfo K., Wongwilai W., Sakai T., Teshima N., Grudpan K. (2020). Determination of albumin, glucose, and creatinine employing a single sequential injection Lab-at-Valve with mono-segmented flow system enabling in-line dilution, in-line single-standard calibration, and in-line standard addition. Molecules.

[14] Kozak J., Paluch J., Kozak M., Duracz M., Wieczorek M., Kościelniak P. (2020). Novel approach to automated flow titration for the determination of Fe(III). Molecules.

[15] Paluch J., Kozak J., Wieczorek M., Woźniakiewicz M., Gołąb M., Półtorak E., Kalinowski S., Kościelniak P. (2020). Novel approach to sample preconcentration by solvent evaporation in flow analysis. Molecules.

[16] Jiménez-López J., Llorent-Martínez E.J., Martínez-Soliño S., Ruiz-Medina A. (2020). Automated photochemically induced method for the quantitation of the neonicotinoid thiacloprid in lettuce. Molecules.

[17] Mantim T., Chaisiwamongkhol K., Uraisin K., Hauser P.C., Wilairat P., Nacapricha D. (2020). Dual-purpose photometric-conductivity detector for simultaneous and sequential measurements in flow analysis. Molecules.

